# Possible antiapoptotic and neuroprotective effects of magnesium sulphate on retina in a preterm hypoxic-ischemic rat model

**DOI:** 10.3906/sag-2012-119

**Published:** 2021-08-30

**Authors:** Serhat İMAMOĞLU, Ebru YALIN İMAMOĞLU, Serkan ERDENÖZ, Alev CUMBUL, Ünal USLU, Şamil AKTAŞ, Fahri OVALI

**Affiliations:** 1 Department of Ophthalmology, Haydarpaşa Numune Training and Research Hospital, University of Health Sciences, İstanbul Turkey; 2 Department of Neonatology, Göztepe Training and Research Hospital, İstanbul Medeniyet University, İstanbul Turkey; 3 Department of Ophthalmology, Okmeydanı Training and Research Hospital, University of Health Sciences, İstanbul Turkey; 4 Department of Histology & Embryology, Faculty of Medicine, Yeditepe University, İstanbul Turkey; 5 Department of Histology & Embryology, Faculty of Medicine, İstanbul Medeniyet University, İstanbul Turkey; 6 Department of Undersea and Hyperbaric Medicine, Faculty of Medicine, İstanbul University, İstanbul Turkey

**Keywords:** Hypoxia-ischemia, magnesium sulfate, neuroprotection, retina

## Abstract

**Background/aim:**

The effects of systemic magnesium sulfate (MgSO_4_) on retina in preterm hypoxic-ischemic (HI) rat model are not known. Our aim was to investigate the effects of MgSO_4_ on retinal ganglion cell (RGC) count, retinal ganglion cell (RGC) apoptotic index, retinal vascular endothelial growth factor receptor-2 (VEGFR-2), and glial fibrillary acidic protein (GFAP) expressions in preterm HI rat model.

**Materials and methods:**

Fifteen, postnatal day (PND) 7 rat pups were divided into 3 groups: 1. Sham-operated group, 2. HI group, and 3. MgSO_4_-treated HI group. The second and third groups underwent ischemia followed by exposure to hypoxia for 2 h (Vannucci model). The first and second groups received intraperitoneal saline and the third group received intraperitoneal MgSO_4_. On PND 10, eyes of the pups were evaluated for RGC count, apoptotic index, VEGFR-2, and GFAP expressions.

**Results:**

In both HI and MgSO_4_-treated HI group, the mean total RGC counts were found to be significantly decreased. However, the mean total RGC count in the MgSO_4_-treated HI group was significantly higher than that of the HI group. The mean apoptotic index was found to be significantly increased in the HI group. Retinal VEGFR-2 and GFAP expressions were found to be significantly higher in the HI group.

**Conclusions:**

Magnesium sulfate preconditioning andtreatment in preterm HI rat model might diminish apoptosis, relatively preserve RGCs, and reduce retinal VEGFR-2 and GFAP expressions.

## 1. Introduction

Neonatal hypoxic ischemic (HI) brain injury is a major cause of morbidity and lifelong disabilities. Approximately 30% of newborns suffering from hypoxia/ischemia do not survive and 20%–40% of the survivors develop chronic neurological impairments [1]. Furthermore, hypoxic insult to the immature retina results in death of retinal ganglion cells (RGCs) and leads to visual impairments in the neonate [2]. The prevalence of visual impairment in children with HI injury ranges from 66%–94%. The visual dysfunctions associated with HI brain injury include strabismus, gaze palsy, nystagmus, optic atrophy, restriction of the visual field, defective color vision, and reduced grating acuity [3]. 

Several experimental models are used to mimic HI in rodents and larger species to study the different categories of injury seen in human infants [4]. Lack of both oxygen (hypoxia) and blood perfusion (ischemia) must be present for a significant period to result in brain injury comparable to that observed in humans [5]. The Vannucci model is one of the most widely used experimental paradigms to induce HI injury in rat pups, resembling HI damage to the human neonatal brain [6]. Huang et al. demonstrated the first evidence of HI retinal damage at both pathological and functional levels using the Vannucci model in neonatal rats [7]. They also suggested that immature retinas are more susceptible to HI injury as compared with those of mature rats.

On exposure to HI insult, both cerebral vascular endothelial growth factor receptor-2 (VEGFR-2) and glial fibrillary acidic protein (GFAP) expressions are known to increase through the activation of hypoxia-inducible factor (HIF)-1α and N-methyl-D-aspartate (NMDA) receptors, respectively [8,9]. However, there is no study in the literature that evaluates the retinal VEGFR-2 and GFAP expressions in the neonatal HI rat model. 

Magnesium sulfate (MgSO_4_) already used in the clinical context might show prophylactic effects during pregnancy, decreasing the incidence of preterm delivery, which in turn reduces the possibility of in utero abnormalities that could lead to HI events [5]. Preclinical evidence suggests that calcium (Ca^2+^) influx blockage via NMDA receptor, thus reducing excitotoxic damage, is the neuroprotective mechanism attributed to MgSO_4 _[10]. Çetinkaya et al., by using the Vannucci model, studied the effects of MgSO_4_ on brains of 7-day-old neonatal rats and demonstrated that MgSO_4_ significantly reduced the percentage of the infarcted brain volume and terminal deoxynucleotidyl transferase (TdT)- mediated deoxyuridine triphosphate (dUTP) nick-end-labeling (TUNEL) positivity [11]. 

Until now, the effects of MgSO_4_ on retina in the neonatal HI rat model has not been studied. Our aim was to investigate the effects of MgSO_4_ on RGC count, RGC apoptotic index, retinal VEGFR-2, and GFAP expressions in the preterm HI rat model.

## 2. Materials and methods

### 2.1. Animals

This study was approved by the Marmara University Animal Care and Use Committee. This study was performed in Yeditepe University, Animal Research Laboratory between June 2019 and August 2019. Dated pregnant Sprague-Dawley rats were housed in individual cages in a temperature-controlled room (21 ± 1 °C) with free access to laboratory chow and water. Rat pups were kept in the same light cycle (lights were on from 7:00 AM to 7:00 PM). Fluorescent lights providing a more natural light environment were used in the laboratory. The pregnant rats were allowed to give birth spontaneously. The day of spontaneous vaginal delivery was considered postnatal day (PND) 1 for the pups. Offspring were reared with their dams until the initiation of the experiments on PND 7. All efforts were made to minimize pain and distress. Rat pups of either sex weighing 10–18 g were used in the study. 

### 2.2. Hypoxic-ischemic injury model and magnesium sulfate treatment

Fifteen rat pups (PND 7) were included in the study. They were considered to have a brain maturity roughly corresponding to that of the human preterm fetal brain (32–34 week-old); that is, the cerebral cortical layering is complete, the germinal matrix is involuting and the white matter is yet to be myelinated [12]. They were divided randomly and equally into 3 groups: Sham-operated (control) group, hypoxic-ischemic (HI) group, and MgSO_4_-treated HI group. 

**Figure 1:**
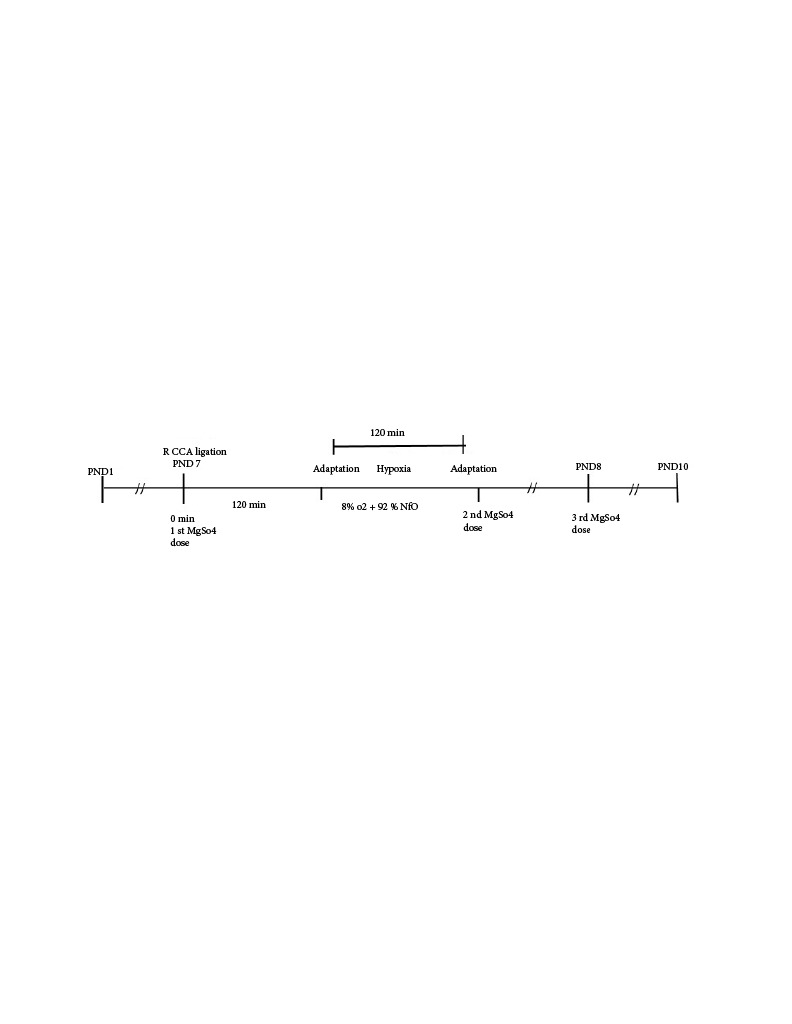
Experimental steps.

Just before ischemia, the first and second groups received intraperitoneal (IP) sterile saline (0.1 mL) and the third group received IP 15% MgSO_4_ (OnPharma, Turkey) (275 mg/kg). The dose of MgSO_4_ used in this study was the same as that used in a previous experimental model of perinatal HI brain injury [11]. This dose was slightly higher than those used in previous experimental and clinical studies [13–15]. We preferred to use 275 mg/kg/dose of MgSO_4_ with the intent of conferring better possible neuroprotection. Magnesium sulfate was chosen in that it might provide preconditioning and neuroprotection at the initial stage of HI. A schematic diagram of the experimental protocol is shown in Figure 1.

To induce HI injury, the Vannucci model was used as previously reported [16]. Animals were anesthetized lightly with inhaled isoflurane (Abbott, Germany). Surgery was performed by two investigators (SE, SI). Under operation microscope (OMS-90 Oakland, NJ), following longitudinal neck incision, the right common carotid artery of the animal was exposed, isolated from the nerve and vein, and sectioned between a double ligature with 5-0 surgical silk. In the sham-operated group, the right common carotid artery was only exposed. Animals exhibiting respiratory arrest due to anesthesia or bleeding during surgery were excluded. All the procedures were performed at room temperature and the total time of surgery never exceeded 3 min. After the closure of the neck incision, pups were allowed to recover for 15 min and returned to their dams for a 2 h recuperation period before HI. The animals other than the sham group were then placed into a chamber partially submerged in 37 °C water to maintain a constant thermal environment and exposed to hypoxia with a warm humidified 8%/92% oxygen/nitrogen mixture for 2 h.

Just after the completion of HI, the second doses of MgSO_4_ and sterile saline were administered intraperitoneally to the MgSO_4_-treated HI group and the HI group, respectively. Following injections, the pups were returned to their cages and allowed to recover for 72 h with their dams. The third dose (24 h after the second dose) was repeated at PND 8 (Figure 1). The sham group also received the second and third doses of IP sterile saline at the same time as other groups.

### 2.3. Sacrifice, cardiac perfusion, and tissue processing 

At PND 10, the rat pups were deeply anesthetized with ketamine (Richter Pharma Ag, Austria) and xylazine (Bayer, Germany) intraperitoneally. Transcardiac perfusion was performed by another investigator (AC). The right eyes of the pups were enucleated and embedded in paraffin wax. Lenses were gently extracted and then the eyes were serially sectioned. 

### 2.4. Estimation of the total number of retinal ganglion cells 

Cell counting was performed by an investigator (UU) blinded to the study groups. To estimate the total RGC count, the optical fractionator technique was used [17]. For quantification of RGCs, the Stereo Investigator v: 11.0 (Micro Bright Field, Colchester VT, USA) was used on a PC system connected to a light microscope (Leica DM 4000B, Wetzlar, Germany). By sampling a subset of the total number of ganglion cell nuclei within the thickness of the retinal tissue and extrapolating, estimates are generated for the total number of RGCs in the entire eye, as described by West et al [18]. 

### 2.5. TUNEL assay and apoptotic index

An in situ cell death detection peroxidase (POD) kit (Roche Diagnostics, Mannheim, Germany) was used for the TUNEL technique and the sections were stained according to the manufacturer’s protocol. In each sampling frame, a counting area was designated without bias and the apoptotic index was calculated by dividing the apoptotic (TUNEL-positive) RGC number by the total RGC number [19]. TUNEL-positive RGCs were counted by the same investigator (UU) blinded to the group assignment.

### 2.6. Immunohistochemical staining and H scoring for vascular endothelial growth factor receptor-2 and glial fibrillary acidic protein

Monoclonal VEGFR-2- specific antibody (Cell signaling, Danvers, MA, USA) and GFAP antibody (Abcam, ab7260, Cambridge, UK) were used for immunohistochemical staining. Immunohistochemical labeling was scored incorporating both the intensity and the distribution of specific staining. H score was derived according to the modification of the previously reported method [20]. It was performed by a single investigator (UU) blinded to the study groups.

### 2.7. Statistical analysis 

Statistical analyses were performed using the SPSS software v: 21.0 (SPSS Inc., Chicago, IL, USA). The variables were investigated using visual (histograms, probability plots) and analytical methods (Shapiro–Wilk’s test) to determine whether or not they are normally distributed. Since the variables are normally distributed, descriptive analyses were presented using mean and standard deviation (SD). One-way ANOVA was used to compare total RGC count, RGC apoptotic index and H scores of VEGFR-2 and GFAP staining between sham-operated, HI and MgSO_4_-treated HI groups. The Levene test was used to assess the homogeneity of the variances. When an overall significance was observed, pairwise posthoc tests were performed using Tukey’s test. An overall p-value of less than 0.05 was considered to show a statistically significant result. 

## 3. Results

Fifteen rat pups were included in the study. The weights of the rat pups were similar for all groups. Total cell counts and apoptotic indices of the RGCs of the study groups were presented in Table 1. In both HI group and MgSO_4_-treated HI group, the mean total RGC counts were found to be significantly decreased when compared to the sham-operated (control) group. However, the mean total RGC count in MgSO_4_-treated HI group was significantly higher than that of HI group. In the HI group, the mean RGC apoptotic index was found to be significantly increased when compared to sham-operated (control) and MgSO_4_-treated HI groups. However, apoptotic indices of MgSO_4_-treated HI group and sham-operated (control) group were similar.

**Table 1:** Total retinal ganglion cell counts and retinal apoptotic indices of the experimental groups.

Groups	Sham-operated(control) (n = 5)	HI (n = 5)	MgSO4-treated HI(n = 5)	p value
Total RGC count	240 ± 98 *,§	158 ± 65 *,†	218 ± 89 §,†	*p: 0.001†p: 0.001§p: 0.013
Apoptotic index	0.02 ± 0.01 ð,¶	0.30 ± 0.02 ð,¥	0.06 ± 0.03 ¶,¥	ðp: 0.001¶p: NS¥p: 0.001

HI: hypoxic-ischemic, MgSO4: magnesium sulfate, RGC: retinal ganglion cell. Total RGC counts were presented as mean ± standard deviation. Apoptotic indices (TUNEL-positive cells/total cells) were presented as mean ± standard deviation. NS: not significant. *, ð =sham vs. HI, §, ¶ =sham vs. MgSO4, †, ¥ =HI vs. MgSO4.

Histological retinal cross-sections for total RGC counting and TUNEL-stained apoptotic RGCs from experimental groups were presented in Figure 2a. 

H scores as an indicator of VEGFR-2 and GFAP expressions in retinal tissue were found to be significantly higher in HI group when compared to other groups (Table 2). On the other hand, H scores for VEGFR-2 and GFAP were similar in sham-operated (control) and MgSO_4_-treated HI groups. As demonstrated in Figure 2b, the retinal layer in HI group had higher VEGFR-2 and GFAP staining intensity in comparison to other groups.

**Figure 2a:**
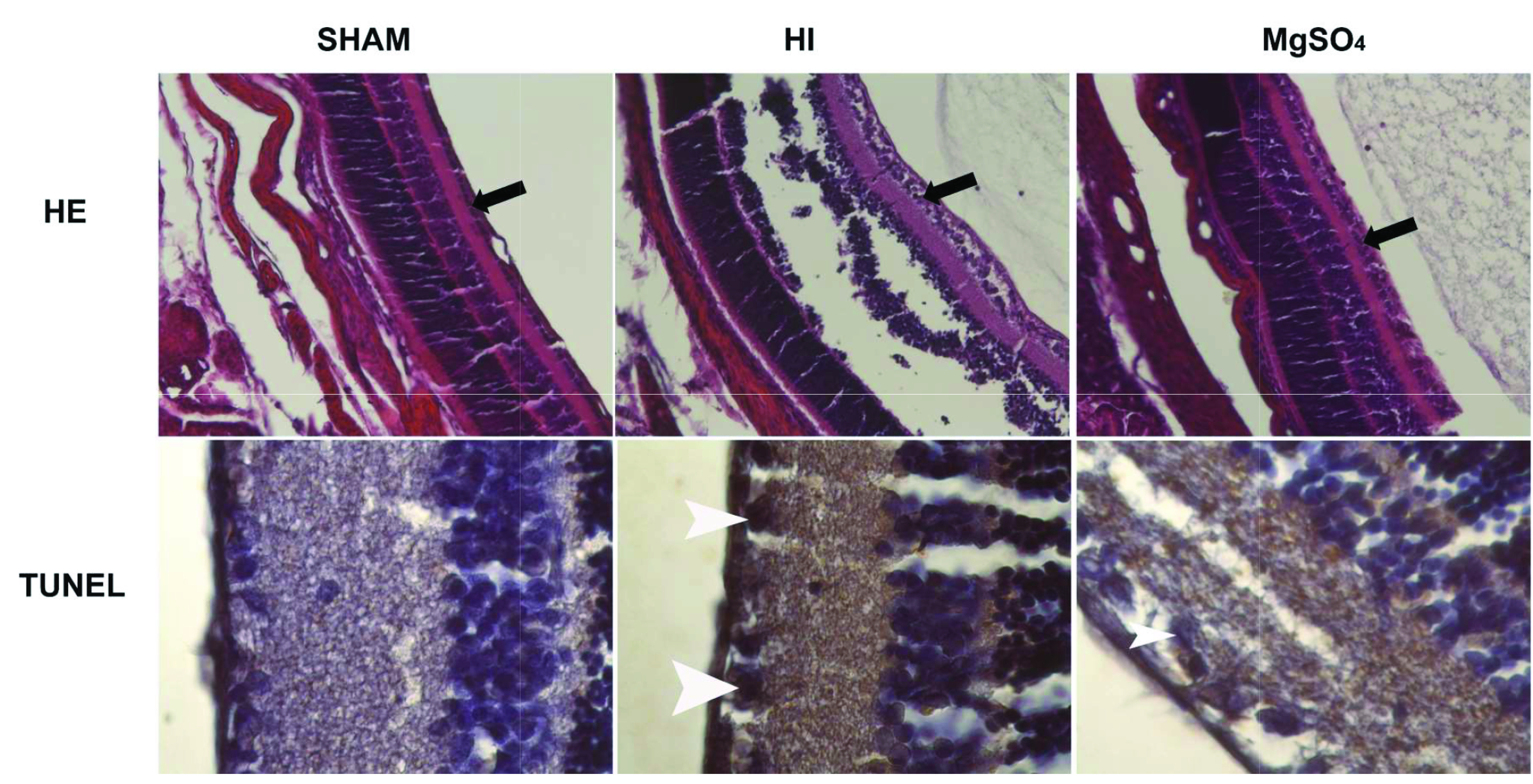
Photomicrographs demonstrating retinal ganglion cells (black arrows) (Hematoxylin and eosin staining, the magnification is x10) and apoptotic retinal ganglion cells (white arrow heads) (TUNEL staining, the magnification is x100) of the rat pups from experimental groups: Sham, HI and MgSO4.

**Figure 2b:**
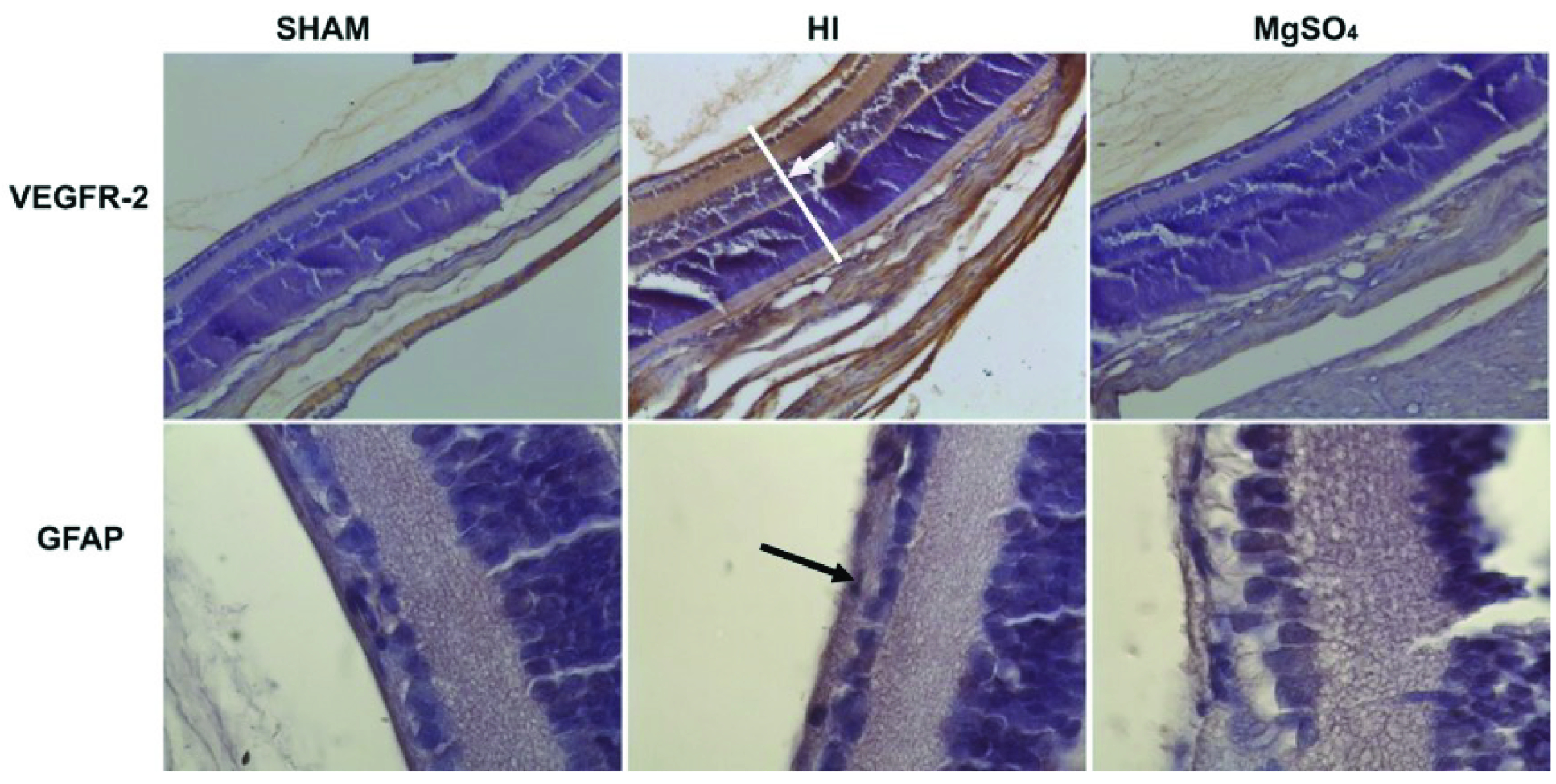
Vascular endothelial growth factor receptor-2 (VEGFR-2) and glial fibrillary acidic protein (GFAP) staining images of the representative retinal sections of the rat pups from experimental groups: Sham, HI and MgSO4. VEGFR-2 staining intensity was found increased in the retina of the HI group (brownish color) (white arrow). The magnification is x20. GFAP staining intensity was found increased in the retina ganglion cell layer of the HI group (brownish color) (black arrow). The magnification is x100

**Table 2:** H scores of experimental groups for vascular endothelial growth factor receptor-2 and glial fibrillary acidic protein.

Groups	Sham-operated(control) *,† (n = 5)	HI *,§(n = 5)	MgSO4-treated HI†,§(n = 5)	p value
H score for VEGFR-2	29.6 ± 5.4	56 ± 4.5	33.6 ± 4.3	*p: 0.001†p: NS§p: 0.001
H score for GFAP	27.4 ± 4.1	56.3 ± 2.3	31.3 ± 4	*p: 0.001†p: NS§p: 0.001

HI: hypoxic-ischemic, MgSO4: magnesium sulfate, VEGFR-2: vascular endothelial growth factor receptor-2, GFAP: glial fibrillary acidic protein. H scores for VEGFR-2 and GFAP were presented as mean ± standard deviation. *=sham vs. HI, p: 0.001, †=sham vs. MgSO4, p: NS (not significant), §=HI vs. MgSO4, p: 0.001.

## 4. Discussion

To our knowledge, this is the first preclinical study evaluating the effects of systemic MgSO_4_ on developing retina after HI insult in neonatal rats. Our results demonstrate that MgSO_4_ might exhibit significant antiapoptotic and neuroprotective effects on the retina in preterm HI rat model by reducing TUNEL-positive cell count, relatively sparing RGCs, and decreasing retinal VEGFR-2 and GFAP expressions.

In this study, the decrease in total RGC count after HI insult in preterm rat retina could be explained by both apoptotic and necrotic RGC death. Although we did not study the necrotic RGC percentages, we observed a significantly increased mean RGC apoptotic index value in the HI group. Even though the mean total RGC count in MgSO_4_-treated HI group was found to be decreased when compared to the control group, the mean apoptotic indices of these two groups were similar. This could be explained by the fact that MgSO_4 _might have a more distinct antiapoptotic effect than its antinecrotic effect on preterm rat retina exposed to HI.

Excess glutamate release in HI conditions causes excitotoxic damage to the RGCs through activation of glutamate (NMDA) receptors [21].Antagonism of NMDA receptors was shown to protect against NMDA-induced retinal cell loss [22].Magnesium, a Ca^2+^ antagonist, is known to antagonize NMDA-mediated excitotoxicity, thereby limiting apoptosis. Magnesium is also essentially and directly involved in mitochondrial membrane stabilization, another mechanism underlying its neuroprotective, antinecrotic, and antiapoptotic effects [23]. Furthermore, in human and animal models, it has been shown that MgSO_4_ increases cerebral blood flow velocities through its vasodilatory effect [24,25]. This increment in cerebral blood flow velocities might explain the protective role of MgSO_4_ in ischemic events and hypoxic brain damage. As a limitation, we could not study the effects of systemic MgSO_4_ treatment on cerebral blood flow in our HI rat model.

Hypoxia-inducible factor-1α is a hypoxia sensor and one of the most predominant effects of HIF- induced transcription is the induction of VEGF and its receptors under hypoxic conditions [8].We also found significantly increased retinal VEGFR-2 expression in PND 7 neonatal rats after HI insult. It is known that VEGF has different effects on different tissues. After cerebral ischemia, the VEGF expressed by microglial cells might have a neurotrophic effect, however the VEGF expressed after retinal ischemia might have a neurodegenerative effect [26,27]. Treatments blocking/decreasing either VEGF (anti-VEGF treatments) or VEGFR-2 expression (such as MgSO_4_) could be neuroprotective in the management of HI retinopathies. 

Müller cells are responsible for the maintenance of homeostasis in the extracellular medium of the retina and protect the neurons by releasing neurotrophic factors. During retinal injury, Müller cells are well known to undergo reactive gliosis characterized by the upregulation of GFAP [28]. Huang et al. demonstrated HI retinal damage using the Vannucci model in preterm neonatal rats and did not find prominent retinal GFAP immunoreactivity until PND 21 [7]. However, in our study, significantly increased retinal GFAP expression, as an indicator of neuronal injury, was detected as early as PND 10 in the HI group. Moreover, Burtrum et al. demonstrated that excitotoxic injury stimulates GFAP expression in PND 7 rat brains [9]. Magnesium sulfate, by antagonizing NMDA receptor-induced GFAP production, seems to decelerate the gliotic response of Müller cells after HI retinal injury, thereby saving time to retinal neurons for reparative processes.

Antenatal MgSO_4_ is strongly recommended for pregnant women at risk of imminent preterm birth before 32 weeks of gestation for fetal neuroprotection [29]. Our experimental model was the ‘preterm’ HI rat model, investigating the possible prophylactic role of systemic MgSO_4_ in preventing both perinatal HI events and retinal injury. We, therefore, administered the first dose of MgSO_4_ just before ischemia. However, most experimental studies have failed to confirm that MgSO_4_ is neuroprotective if given immediately before, during or after HI [30,31]. Koning et al. demonstrated that via providing mitochondrial protection, MgSO_4 _induces strong preconditioning, i.e*.* it reduces the vulnerability of the immature brain to a subsequent severe insult when administered 6 days to 12 h before the induction of HI in the PND 7 rat [32].The MgSO_4_ dose was 1.1 g/kg as a bolus in that study, whereas we administered 275 mg/kg/dose as three doses, the first just before ischemia and the remaining two after hypoxia. The total MgSO_4_ dose was less (three-fourths) than that of Koning’s study. As an extension of the brain, the retinas of PND 7 rats in our study were found to be relatively spared after HI insult, even though MgSO_4 _was administered just before HI. Moreover, we unfortunately could not investigate the dose-dependent effect of MgSO_4_ on retina in this preterm HI rat model. This was a limitation of our study.

A common hallmark in retinal ischemia models is the progressive degeneration and final neuronal loss in the ganglion cell layer (GCL) [7]. Therefore, we focussed on GCL in our study. We did not evaluate the other retinal (inner plexiform, inner nuclear, outer plexiform, and outer nuclear) layers and optic nerve at the pathological level. Furthermore, we could not study HI retinal damage and effects of MgSO_4_ treatment at the functional level with electroretinography. Our results might reflect only short-term effects of MgSO_4_ on retinas of HI rat pups. We did not study the long-term effects. Also, the results of this experimental study could not be totally applied on human beings. These are limitations of our study.

In conclusion, systemic MgSO_4 _preconditioning and treatment in the preterm HI rat model might diminish apoptosis, relatively preserve RGCs, and reduce HI-induced retinal VEGFR-2 and GFAP expressions, suggesting that it could be a therapeutic agent in attenuating the HI insult to the developing retina. 

## Informed consent

This study was approved by the Marmara University Animal Care and Use Committee (protocol number: 32.2014.mar).
